# Anaphylactic reactions in the build-up phase of rush immunotherapy for bee venom allergy in pediatric patients: a single-center experience

**DOI:** 10.1186/s12948-022-00170-3

**Published:** 2022-04-29

**Authors:** Antonia Glaeser, Christoph Müller, Sebastian Bode

**Affiliations:** 1 Center for Pediatrics and Adolescent Medicine, University Medical Centre and Faculty of Medicine Freiburg, Freiburg, Germany; 2 Department of Pediatrics and Adolescent Medicine, Ulm University Medical Center, Ulm, Germany

## Abstract

**Background:**

Anaphylaxis occurs in up to 3.5% of hymenoptera stings and can be a life-threatening emergency. Venom immunotherapy (VIT) provides excellent protection from further episodes of anaphylaxis and is well tolerated. In this study the frequency of anaphylactic reactions in pediatric patients undergoing rush bee venom immunotherapy was assessed as well as possible risk factors and modified up-dosing schemes are reported.

**Methods:**

19 consecutive pediatric patients, who had previously experienced an anaphylactic reaction following a bee sting and showed IgE-mediated sensitization to bee venom, underwent inpatient rush immunotherapy with bee venom extract. We retrospectively compared serological findings (total IgE, serum tryptase level, sensitization to Api m1, Api m3 and Api m10 bee venom allergens) and possible risk factors between patients who experienced an anaphylactic reaction during immunotherapy and patients who did not.

**Results:**

Three of the included 19 patients (15.8%) developed anaphylactic reactions to rush bee venom immunotherapy, all of them between administration of 40 and 80 µg of bee venom extract. However, all three patients reached the standard maintenance dose of 100 µg of bee venom following a modified VIT schedule without any further complications. Total serum IgE levels as well as Api m3 sensitization levels were significantly higher in patients showing an adverse reaction to bee VIT compared to those who did not experience any complications. There were no statistically significant differences concerning age, pre-existing conditions, type and severity of the initial reaction and Api m1, Api m10 and serum tryptase levels between the two subgroups.

**Conclusion:**

Even if anaphylactic reactions occur during the build-up phase of VIT for bee venom in children and adolescents, venom immunotherapy can and should be continued in most cases.

## Introduction

Anaphylaxis, an acute IgE-mediated systemic allergic reaction, affects 7–50 in 100,000 people a year [1–3] and accounts for up to 1% of presentations in emergency rooms [4]. Hymenoptera stings, including honeybees (*Apis mellifera*) and certain species of wasp (family Vespidae), are the most common trigger for anaphylactic reactions in adults (55%), and the second most common trigger in children (24%) [5]. Anaphylaxis occurs in up to 3.5% of bee or wasp stings and can be a life-threatening emergency requiring immediate treatment [6]. While the most typical manifestation of anaphylaxis caused by insect stings is a generalized cutaneous reaction and most patients recover without any permanent sequelae, airway obstruction and cardiovascular involvement can be fatal [7].

The only causal treatment for allergy against hymenoptera venom is venom immunotherapy (VIT). VIT can induce tolerance to hymenoptera venom through administration of gradually increasing quantities of the relevant allergens. VIT is indicated in all pediatric patients who have experienced a severe generalized anaphylactic reaction (Grade 2 and higher on the Ring and Messmer grading scale [8]) and after mild generalized reactions (Grade 1), if the causative allergen cannot be avoided due to high exposure risk or if the application of the prescribed anaphylaxis emergency kit proves to be problematic [6].

Among different VIT regimes rush protocols have been established as the method of choice as they provide fast protection against further hymenoptera stings and have been shown to improve the efficiency of immunotherapy [9]. Once the standard maintenance dose of 100 µg of insect venom is reached, VIT needs to be continued for at least three to five years. After discontinuation, ongoing protection against further anaphylactic reactions can be expected with an efficacy of 77–84% for honeybee venom compared to 91–96% for vespid venom [6, 10, 11].

Adverse effects to VIT mostly occur during the build-up phase of venom immunotherapy [12]. Localized reactions such as reddening, swelling, itching and tenderness around the injection site can be expected in roughly one third of pediatric patients [12]. However, 3.1 to 50% of patients undergoing VIT for bee or wasp venom allergy experience severe systemic reactions similar to those following natural allergen exposure—regardless of age [13–16]. Anaphylactic reactions during VIT are more common in bee venom than in wasp VIT [13, 17, 18]. Some studies show a higher risk for systemic reactions to bee VIT if a build-up phase with rapid dose increments is used [19]. Others have found no differences between pediatric patients receiving (ultra-) rush or conventional venom immunotherapy [9] or could even show a higher risk for slower build-up phase protocols [17]. Risk factors for systemic reactions during hymenoptera VIT include current infections, allergic symptoms, mastocytosis and/or an elevated basal serum tryptase concentration as well as pre-existing conditions as hyperthyroidism or insufficiently treated asthma as well as co-medication with beta blockers [6, 12, 20–22].

There is a paucity of data on pediatric patients concerning the significance of the severity of former reactions and of serological findings such as IgE levels in predicting an anaphylactic reaction to hymenoptera VIT. In some studies, both high [23] and low [24] rApi m1 and high rApi m4 [23] sensitization levels have been identified as possible risk factors, while others did not show any correlation between serological results and the risk of anaphylaxis.

The purpose of this study was to assess the frequency of anaphylactic reactions in pediatric patients undergoing rush bee VIT at our institution. We aimed to identify possible risk factors in clinical history or serological markers for anaphylactic side effects of rush up dosing of bee VIT in children.

## Methods

### Study design and study cohort

In total, 19 consecutive patients who presented to our institution between January 2020 and December 2020 for inpatient rush immunotherapy with bee venom were included in this single-center retrospective study.

All patients had experienced an anaphylactic reaction following a bee sting. The severity of this initial anaphylaxis was graded according to the Ring and Messmer grading scale for anaphylactic reactions and dermal, gastrointestinal, respiratory and cardiovascular manifestations were recorded [6, 8].

All patients included in this study underwent serological testing for total IgE and serum tryptase levels as well as Api m1, Api m3 and Api m10 bee venom allergens and showed IgE-mediated sensitization to bee venom.

### Protocol for bee venom rush immunotherapy

All patients were free of infectious symptoms before start of immunotherapy and were nil by mouth for at least 6 h.

Rush Immunotherapy was carried out using *ALK- lyophilized SQ*^*®*^ bee venom with an allergen content of 100,000 SQ units/ml (equals 100 µg/ml). Table 1 illustrates the rush protocol used. During rush-immunotherapy oxygen saturation was continuously monitored, blood pressure and pulse were assessed every 5 min. After a 2-h observation period after the last injection patients were discharged home on the third day of the protocol.

**Table 1:** Rush protocol using *ALK-lyophilized SQ*^*®*^ bee venom. The dose of lyophilized bee venom injected subcutaneously is indicated in μg in the right column

Day 1	Dose administered
Minute 0	1 μg
Minute 30	10 μg
Minute 60	20 μg
Day 2	
Minute 0	20 μg
Minute 30	40 μg
Minute 60	80 μg
Day 3	
Minute 0	100 μg

If side effects occurred at any point during the protocol, further proceedings were decided on a case-by-case basis by the pediatric allergist in charge. All anaphylactic side effects were classified according to the Ring and Messmer grading scale for anaphylactic reactions [6, 8].

### Statistical analysis

Comparisons between the two subgroups of patients without side effects and those experiencing anaphylactic side effects of immunotherapy were carried out using Fisher’s exact test for categorical data, the Mann–Whitney U test for ordinal data and using the unpaired two-sample Student’s t test for continuous data.

Since this was an explorative analysis, the resulting p-values were not adjusted for multiplicity and thus have no confirmatory value. P values < 0.05 were regarded as statistically significant. A Multivariate analysis was not performed due to the small number of patients.

## Results

### Patient characteristics

Table 2 illustrates characteristics of the study cohort. In total, 19 pediatric patients who had undergone rush immunotherapy with bee venom at our institution in 2020 were included. Four of the included patients (21.1%) had relevant pre-existing conditions. Two patients suffered from autoimmune diseases (juvenile arthritis and PFAPA (Periodic fever, aphthous stomatitis, pharyngitis and adenitis) syndrome), one patient had a dust mite allergy and one patient had been treated for myelodysplastic syndrome with stem cell transplantation. Most patients had shown grade II or III anaphylactic reactions to the initial field bee sting. Three patients with anaphylaxis grade I had additional risk factors (e.g., bee keeper in family) and indication for VIT was established in shared decision making between caregivers and pediatric allergists according to national guidelines [6].

**Table 2:** Study cohort

Variable	*N*	(%)
No. of patients	19	
Age in years		
Median	9	
Minimum	4	
Maximum	14	
Pre-existing conditions	4	(21.1%)
Other allergies	1	
Autoimmune	2	
Hematological	1	
Grade of anaphylactic reaction to initial field sting		
Grade I	3	
Grade II	8	
Grade III	8	
Grade IV	0	
Anaphylactic reaction to rush immunotherapy	3	(15.8%)
Grade I	1	
Grade II	2	
Grade III	0	
Grade IV	0	

10/17 (58.8%) of patients had local erythema or swelling that resolved spontaneously. Three of the included 19 patients (15.8%) showed an anaphylactic reaction to rush bee venom immunotherapy. All anaphylactic reactions occurred on day two of the rush protocol. Two patients developed an anaphylactic reaction after 80 μg of bee venom was given subcutaneously and the other patient reacted after the administration of 40 μg of bee venom. One of the patients had a grade I reaction with generalized urticaria and the two other patients experienced grade II reactions with urticaria and coughing in one and dyspnea in the other case.

Both patients experiencing grade II reactions had preexisting conditions (PFAPA syndrome and juvenile arthritis in the other patient), while for two of the 16 patients without complications preexisting conditions were recorded (myelodysplastic syndrome treated with allogenic stem cell transplantation and dust mite allergy).

Furthermore, there were no significant differences concerning the patients’ age and the type and severity of the initial reaction or possible co-anaphylactic triggers (Table 3) between the patients experiencing an adverse reaction to immunotherapy and the group without complications.

**Table 3:** Initial anaphylactic reaction to a bee field sting compared between patients experiencing an adverse reaction (AR) to immunotherapy and those without complications (NC)

	All patients	AR	NC	
No. of patients	19	3	16	*P*
Median grade of anaphylactic reaction to initial field sting	2	2	2	n.s
No. (%) of patients with dermal symptoms	19 (100.0)	3 (100.0)	16 (100.0)	n.s
(95% CI)	(100 to 100)	(100 to 100)	(100 to 100)	
No. (%) of patients with gastrointestinal symptoms	6 (31.6)	1 (33.3)	5 (31.3)	n.s
(95% CI)	(12.6 to 56.6)	(0.8 to 90.6)	(11.0 to 58.7)	
No. (%) of patients with respiratory symptoms	13 (68.4)	2 (66.7)	11 (68.8)	n.s
(95% CI)	(43.5 to 87.4)	(9.4 to 99.2)	(41.3 to 89.0)	
No. (%) of patients with cardiovascular symptoms	10 (52.6)	1 (33.3)	9 (56.3)	n.s
(95% CI)	(28.9 to 75.6)	(0.8 to 90.6)	(29.9 to 80.3)	

*AR* patients experiencing an adverse reaction to immunotherapy, *NC* patients undergoing immunotherapy without complications, *CI* confidence interval, *No.* number

### Comparison of clinical and serological findings between patients experiencing an adverse reaction to immunotherapy and those without complications

Neither the grade of the anaphylactic reaction to the initial field bee sting, nor the occurrence of dermal, gastrointestinal, respiratory or cardiovascular symptoms were significantly different in patients experiencing an adverse reaction to rush venom immunotherapy compared to those without any complications (Table 3).

As Table 4 illustrates, total serum IgE levels and Api m3 sensitization levels were significantly higher in patients showing an adverse reaction (AR) to bee venom immunotherapy compared to those who did not experience any complications (NC). Api m1, Api m10 and serum tryptase levels did not differ significantly between the two subgroups.

**Table 4:** Comparison of the serological findings between patients experiencing an adverse reaction (AR) to immunotherapy and those without complications (NC)

	All patients	AR	NC	
No. of patients	19	3	16	*P*
sIgE honey bee venom [kU/l] m (± SD)	*(n* = *19)* 38.1 (± 36.3)	*(n* = *3)* 82.7 (± 15.3)	*(n* = *16)* 29.7 (± 14.0)	0.019*
Api m1 [kU/l] m (± SD)	*(n* = *17)* 13.8 (± 24.3)	*(n* = *3)* 29.3 (± 10.2)	*(n* = *14)* 10.5 (± 14.0)	0.253
Api m3 [kU/l] m (± SD)	*(n* = *16)* 8.4 (± 7.9)	*(n* = *2)* 22.3 (± 1.9)	*(n* = *14)* 6.4 (± 8.9)	0.005**
Api m10 [kU/l] m (± SD)	*(n* = *18)* 20.2 (± 33.5)	*(n* = *3)* 38.6 (± 43.5)	*(n* = *15)* 16.5 (± 35.1)	0.326
Serum tryptase [μg/l] m (± SD)	*(n* = *14)* 3.6 (± 2.6)	*(n* = *1)* 6.2 (± 0)	*(n* = *13)* 3.4 (± 2.2)	0.348

For the different groups the respective averages, the minimum, the maximum and the standard deviation for total IgE, Api m1, Api m3, Api m10 are indicated. The units are indicated in square brackets. *p < 0.05, **p < 0.01, ***p < 0.001

*AR* patients experiencing an adverse reaction to immunotherapy, *NC* patients undergoing immunotherapy without complications, *No.* number, *SD* standard deviation, *m* mean

### Continuation of immunotherapy in patients having shown an adverse reaction to bee venom rush immunotherapy

In all three patients who had shown an anaphylactic reaction during rush venom immunotherapy, emergency medication (antihistamines, corticosteroids and fluids) was administered immediately and the immunotherapy was stopped. None of the included patients needed treatment with epinephrine or beta-adrenergic receptor agonists as symptoms resolved quickly.

In one patient, on the day following the grade I anaphylactic reaction, bee venom doses of 40 μg, 80 μg and 100 μg were administered, thereby concluding the build-up phase. Ninety minutes after the administration of 100 μg of bee venom, the patient developed a dry cough without suspicious auscultatory findings and without impact on monitoring. The symptom resolved spontaneously and did not require intervention.

The two remaining patients with grade II anaphylactic reactions after build-up phase were readmitted after 3 and 4 weeks respectively and subsequently underwent an adapted build-up phase. Over three days the bee venom dose was increased to 60 µg in an inpatient setting. Three to five days later, we repeated the dose of 60 µg once in an outpatient setting and then further increased the bee venom dose to 80 µg and 100 µg at one-week intervals. One week after this modified build-up phase, the maintenance dose of 100 µg of bee venom was repeated once. We did not observe any anaphylactic reactions during this specifically tailored build-up phase. All three patients reached the standard maintenance dose of 100 µg of bee venom and subsequently entered the maintenance phase involving monthly injections. No further anaphylactic reactions were reported during the first two years of maintenance therapy.

## Discussion

In this study, we aimed to assess the frequency of anaphylactic reactions in pediatric patients undergoing the build-up phase of rush bee venom immunotherapy and to identify possible risk factors in a small, but representative pediatric cohort in a pediatric university hospital. Due to the small number of included patients and only three anaphylactic reactions no generalizable conclusions should be drawn from this study—but some interesting findings should be noted.

We found that three of the included 19 patients (15.8%) developed an anaphylactic reaction to rush bee venom immunotherapy. In previous studies, the incidence of severe systemic reactions to bee or wasp VIT in adult patients varies from 3.1 to 50% [13–16]. A systematic review found that the evidence of systemic reactions to bee VIT was 25.1% [18]. All studies use slightly different updosing regimes and the populations studied are mainly adults. While the frequency of anaphylactic reactions to bee VIT in our small pediatric cohorts is smaller compared to the systematic review, it cannot be generalized whether this might be due to the small cohort, the pediatric patients studied or the rush protocol that was used. Overall, our result seems to be consistent with previous literature.

Two of the three patients with anaphylactic reactions suffered from immunological conditions (PFAPA syndrome and juvenile arthritis). Whether these conditions may pose an increased risk for anaphylaxis during VIT through continuous over-activation of immunological pathways is not fully understood and as of now these conditions, if controlled, are not a contraindication for VIT [20]. The cohort reported is to small to draw generalizable conclusions.

In our cohort, all anaphylactic reactions occurred after administration of a dose of 40–80 μg of bee venom. Systemic reactions to bee venom immunotherapy have been reported for doses from 1 to 100 μg [25, 26] During a honeybee sting an average dose of 59 μg of bee venom is injected, which is comparable to the doses that caused anaphylactic reactions in our cohort [27].

We found that total serum IgE levels and Api m3 sensitization levels were significantly higher in patients showing an adverse reaction to bee venom immunotherapy compared to those who did not experience any complications.

Contrary to earlier studies [20–24], we found that Api m1, Api m10 and serum tryptase levels did not differ significantly between the two subgroups. Data regarding the role of Api m3 sensitization and anaphylactic reactions during VIT is lacking in the literature. There is evidence that Api m3 might be underrepresented in therapeutic extracts [28], but this should not influence the clinical course in sensitized individuals. The severity of former reactions to bee stings did not seem to predispose patients to anaphylactic side effects, as previously been suggested in the literature [24].

Whether this is due to the small number of patients in this study or represents a typical pediatric cohort remains to be elucidated and no generalizable conclusions should be drawn from these findings.

The most important finding of this study is that even if anaphylactic reactions occur during build-up phase all patients in our small cohort could continue VIT and reach the maintenance dose with an adapted regimen. This is in accordance with previous studies [17] and supports the approach that all pediatric patients who have experienced a severe generalized anaphylactic reaction to bee venom can and should complete VIT—even if an adverse reaction occurs during the build-up phase—as VIT is the only causal treatment option for hymenoptera allergy and offers reliable protection against further anaphylactic episodes.

## Conclusion

Rush venom immunotherapy against hymenoptera venom is safe in pediatric patients and was safe in this small retrospective cohort study. As of now there are no definitive predictive serological parameters that can help to identify patients that may be at risk for developing anaphylactic reactions during rush immunotherapy. The risk of anaphylactic reactions however seems to be elevated in the dosing interval between 40 and 80 μg—the same amount of venom that is administered by a field sting. Even patients who developed anaphylactic reactions during build-up phase could continue VIT with a modified up-dosing protocol and did not experience further side-effects.

## Data Availability

All data generated or analyzed during this study are included in this published article.

## Abbreviations

AR: Adverse reaction
CI: Confidence interval
Ig: Immunoglobulin
m: Mean
NC: No complications
No: Number
PFAPA: Periodic fever, aphthous stomatitis, pharyngitis and adenitis
SD: Standard deviation
VIT: Venom immunotherapy
